# Warm aortic arch repair: A new approach

**DOI:** 10.1016/j.xjtc.2025.01.003

**Published:** 2025-01-23

**Authors:** Shinichi Fukuhara, Robert B. Hawkins, Gorav Ailawadi, Barbara Hamilton

**Affiliations:** Department of Cardiac Surgery, University of Michigan, Ann Arbor, Mich

**Keywords:** hemiarch aortic repair, partial aortic arch repair, hypothermic circulatory arrest, antegrade cerebral perfusion

## Abstract

**Objectives:**

We present a streamlined approach for aortic arch repair that does not require hypothermic circulatory arrest or axillary/femoral cutdown.

**Methods:**

The procedure setup comprised standard right radial/femoral arterial lines, near-infrared spectroscopy, sternotomy, and cardiopulmonary bypass with arch central cannulation. Under normothermia, antegrade cerebral perfusion (ACP) was administered through the innominate artery via punctured aortic root needle cannula (closed ACP) or balloon catheter (open ACP). Aortic arch clamping followed, with bilateral ACP employed in selected cases. From 2019 to 2024, a total of 153 patients, including 48 (30.4%) with Type A aortic dissection, underwent warm arch repair.

**Results:**

The majority of repairs involved hemiarch (n = 137 [89.5%]), with a smaller subset of patients with zone 1 (n = 5 [3.3%]) and zone 2 (n = 11 [7.2%]) arch repair. The volume of warm arch repair cases increased during the study period, with its establishment as the standard approach since 2023, regardless of aortic pathologies. Median ACP flow rates were 8.6 mL/kg/minute and 11.3 mL/kg/minute for unilateral and bilateral ACP, respectively, with a technical success rate of 99.4%. In-hospital mortality and disabling stroke were 2.0% and 1.3%, respectively. Since the launch of this approach and initial experience, 4 surgeons at our institution have adopted this technique and the clinical indications for this approach have evolved.

**Conclusions:**

Warm aortic arch repair without hypothermic circulatory arrest or axillary/femoral cutdown is demonstrated to be safe, feasible, and reproducible. It has emerged as a new and valid approach for various aortic pathologies requiring hemiarch and selected partial aortic arch repair.

**Figure:**
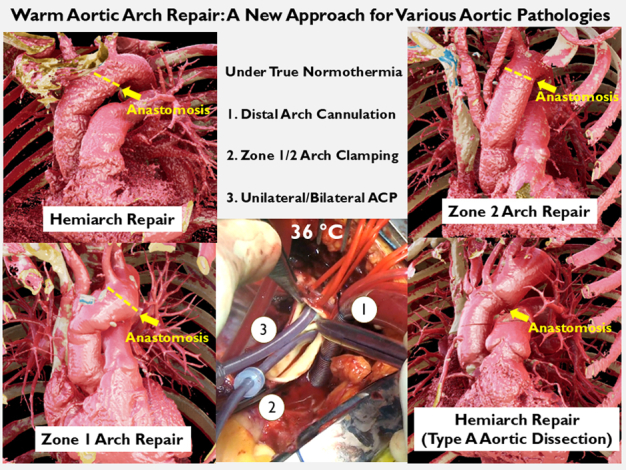
Warm aortic arch repair with various clinical settings.

**Central Message**

Warm aortic arch repair without hypothermic circulatory arrest or axillary/femoral cutdown is demonstrated to be safe and feasible, emerging as a new and valid approach for various aortic pathologies.

**Perspective**

We introduced warm aortic arch repair, which eliminates the necessity for hypothermic circulatory arrest or axillary/femoral cutdown in selected cases, resulting in reduced operative time and favorable clinical outcomes. This technique is safe, feasible, and reproducible, thus emerging as a new and valid approach for various aortic pathologies requiring hemiarch and selected partial aortic arch repair.

See Commentator Discussion on page 32.

Historically, recognized advancements in aortic arch surgery include the introduction of hypothermic circulatory arrest^1^ followed by the development adjunctive cerebral perfusion strategy utilizing retrograde and antegrade cerebral perfusion (ACP).^2^^,^^3^ Despite these evolving techniques, numerous questions persist: What is the optimal body temperature during circulatory arrest? and, What represents the ideal cerebral perfusion strategy? To date, consensus on neuroprotection strategies during arch repair remains elusive. Additionally, the clinical influence of neurological injury and mortality after arch repair remains substantial, approaching 11% and 26% of combined complication rates in non-aortic dissection and aortic dissection cohorts, respectively.^4^^,^^5^ The inherent risks associated with hypothermic circulatory arrest continue to be of remarkable concern despite contemporary refinements in surgical techniques.

In this context, a novel streamlined approach, the warm aortic arch repair, that obviates the need for hypothermic circulatory arrest and axillary or femoral cannulation, has been implemented in our practice since 2019. Initially utilized for simple aneurysms in the early stages of implementation, the clinical indications and the extent of arch repair have since broadened. Presently, warm arch repair has evolved through accrued experiences and the learning curve to emerge as a new routine approach for various pathologies requiring hemiarch and selected partial arch repair. The aim of this study is to share insights and lessons learned from our initial experiences.

## Methods

The University of Michigan Institutional Review Board approved all aspects of the study (HUM00133791; approved December 1, 2017). The approval included a waiver of informed consent.

### Patients and Study Design

Between November 2019 and March 2024, 153 patients underwent warm aortic arch repair. Among them, 48 (30.4%) patients had Type A aortic dissection, comprising 32 (66.7%) DeBakey type 1 and 16 (33.3%) DeBakey type 2 dissections. The extent of arch repair was as follows: hemiarch (n = 137 [89.5%]), zone 1 (n = 5 [3.3%]), and zone 2 (n = 11 [7.2%]) repair. The clinical characteristics and outcomes between patients with and without Type A aortic dissection were compared. Investigators used prospectively maintained clinical data regarding aortic pathologies and operative details. Additionally, the Society of Thoracic Surgeons data elements from the University of Michigan Cardiac Surgery Data Warehouse were retrieved for standard perioperative variables, including preoperative, intraoperative, and postoperative characteristics. The National Death Index database and Michigan Death Index Database were used, as well as electronic medical record review to obtain data on long-term survival.

### Surgical Technique

Video 1 demonstrates the conduct of the operation. The standard procedure setup included arterial lines for the upper (right radial artery) and lower body (femoral artery), near-infrared spectroscopy, sternotomy, and cardiopulmonary bypass institution with central aortic arch cannulation and standard venous cannulation. Normothermia (36 °C) was maintained using heater-cooler system, rather than drifting the temperature, in all cases.

#### Aortic arch cannulation

**Figure 1:**
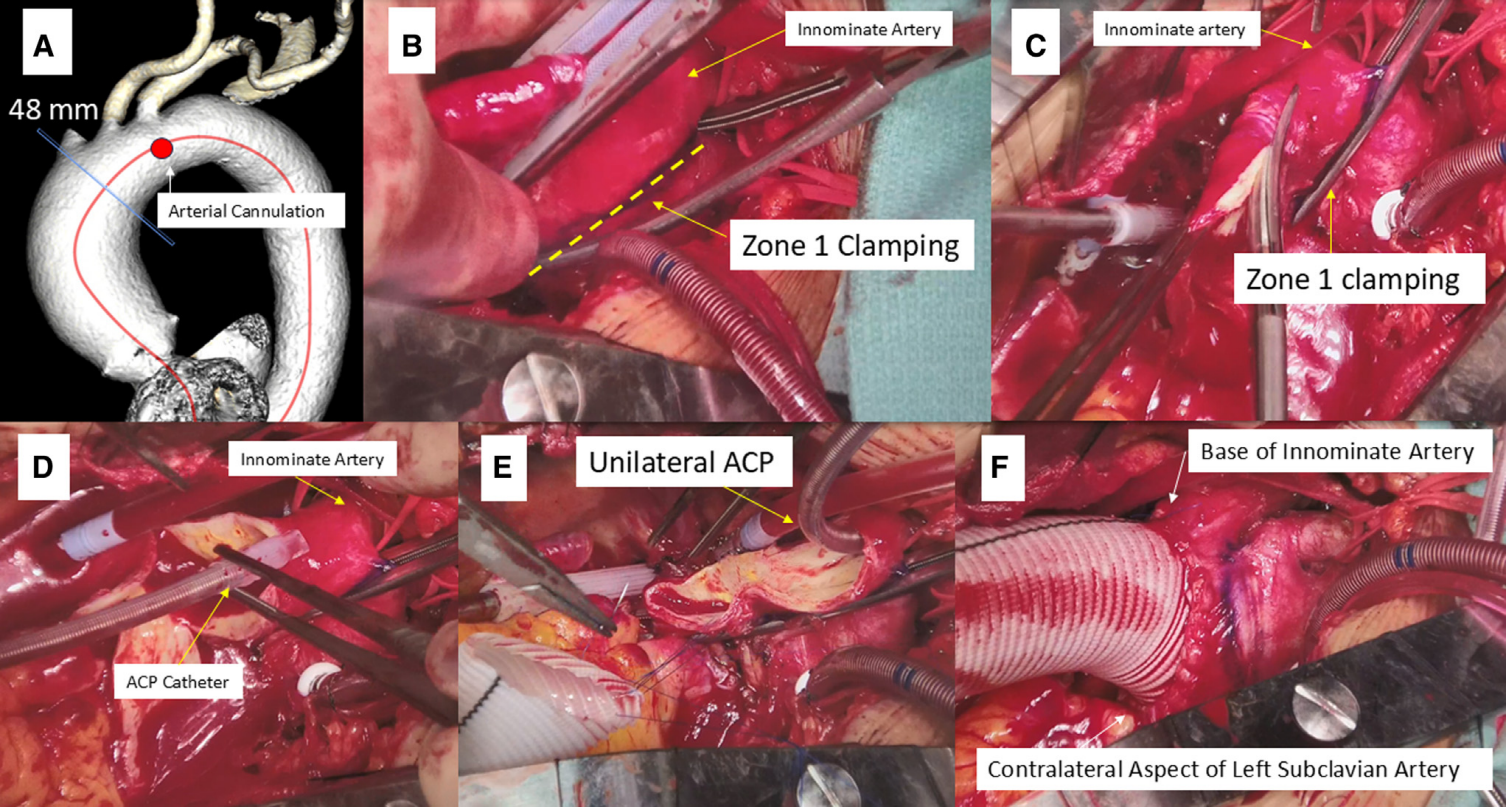
Warm hemiarch repair utilizing unilateral antegrade cerebral perfusion (*ACP*). A, Arterial cannulation site. B, Aortic arch clamping at zone 1. C, Opening the distal ascending aorta before ACP catheter insertion. D, ACP catheter insertion. E, Distal anastomosis after establishment of unilateral ACP. F, Distal anastomosis completion.

Cannulation was performed at the anterosuperior aspect of the arch between the left common carotid artery and left subclavian artery (Figure 1, *A*) after placing double purse-string sutures. Extreme caution was used not to injure the left recurrent laryngeal nerve. Generally, cannulation along the lessor curvature of the arch is unsuitable due to interference with aortic arch clamping. For cases of DeBakey type 1 aortic dissection, an arterial cannula was inserted into the true lumen using the Seldinger technique under transesophageal echocardiography guidance.

#### ACP administration

**Figure 2:**
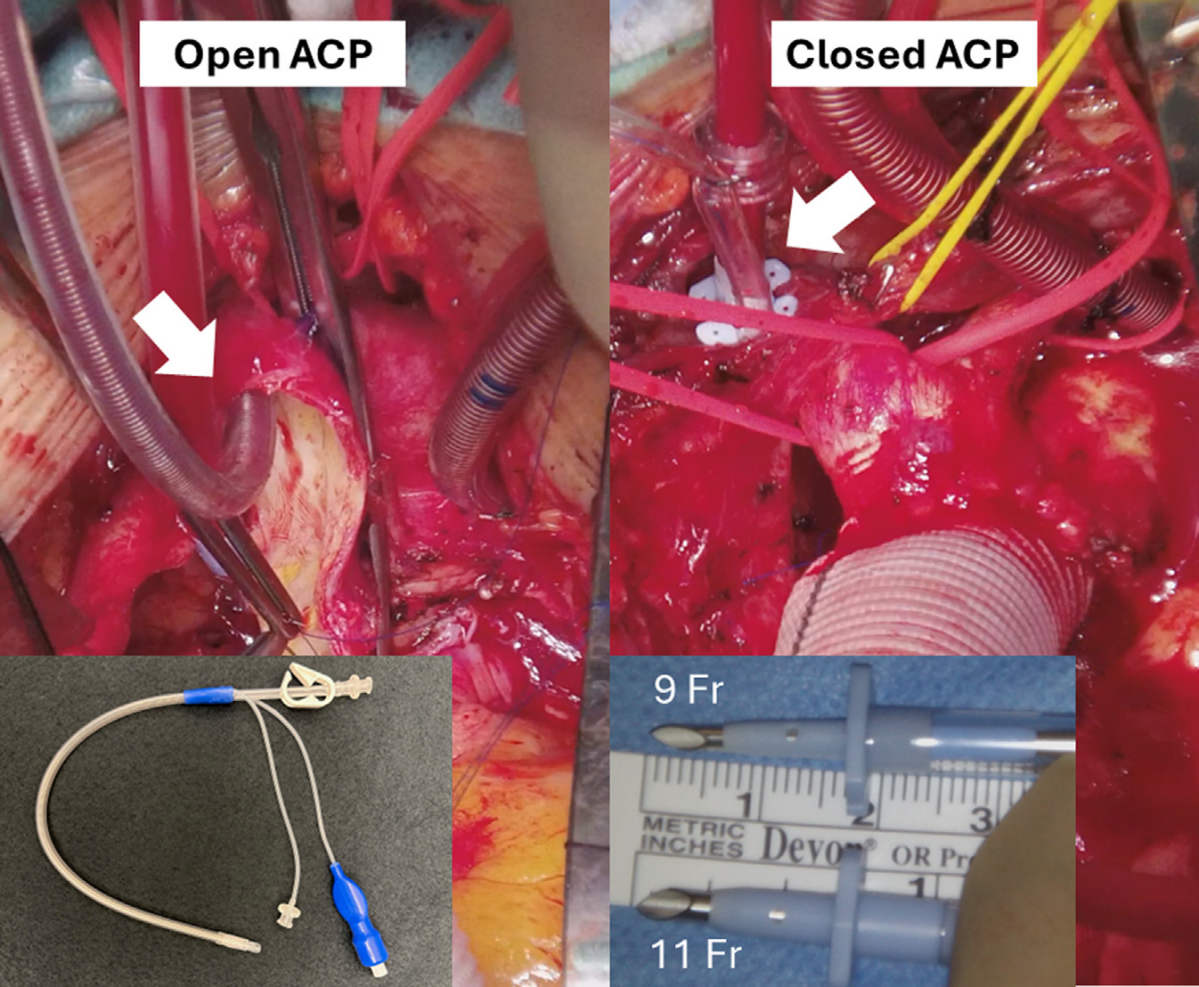
Open and closed antegrade cerebral perfusion (*ACP*).

The innominate and left carotid arteries were mobilized, and a vessel loop was doubly encircled around the innominate artery. The ascending aorta was crossclamped and myocardial protection was achieved in the standard fashion. Irrespective of concomitant procedures being performed, the arch repair part was performed first. ACP delivery methods consisted of closed ACP and open ACP (Figure 2). The ACP flow was determined to maintain right radial mean arterial pressure within the range of 50 to 80 mm Hg. The ACP perfusate temperature was also 36 °C.

##### Closed ACP

Early in the series, warm arch repair was conducted utilizing the closed ACP. ACP was administered through the innominate artery via a punctured aortic root needle cannula (DLP; Medtronic Inc). A 5-0 polypropylene *U* stitch was placed on the anterior aspect of the innominate artery, and the cannula was placed. Unilateral ACP was then delivered after crossclamping the base of the innominate artery, with verification of adequate right radial arterial pressure before arch clamping. Although the closed ACP approach is still applied in selected cases with a bovine trunk, allowing bilateral ACP from a single cannula, it has predominantly been supplanted by the open ACP method.

##### Open ACP

**Figure 3:**
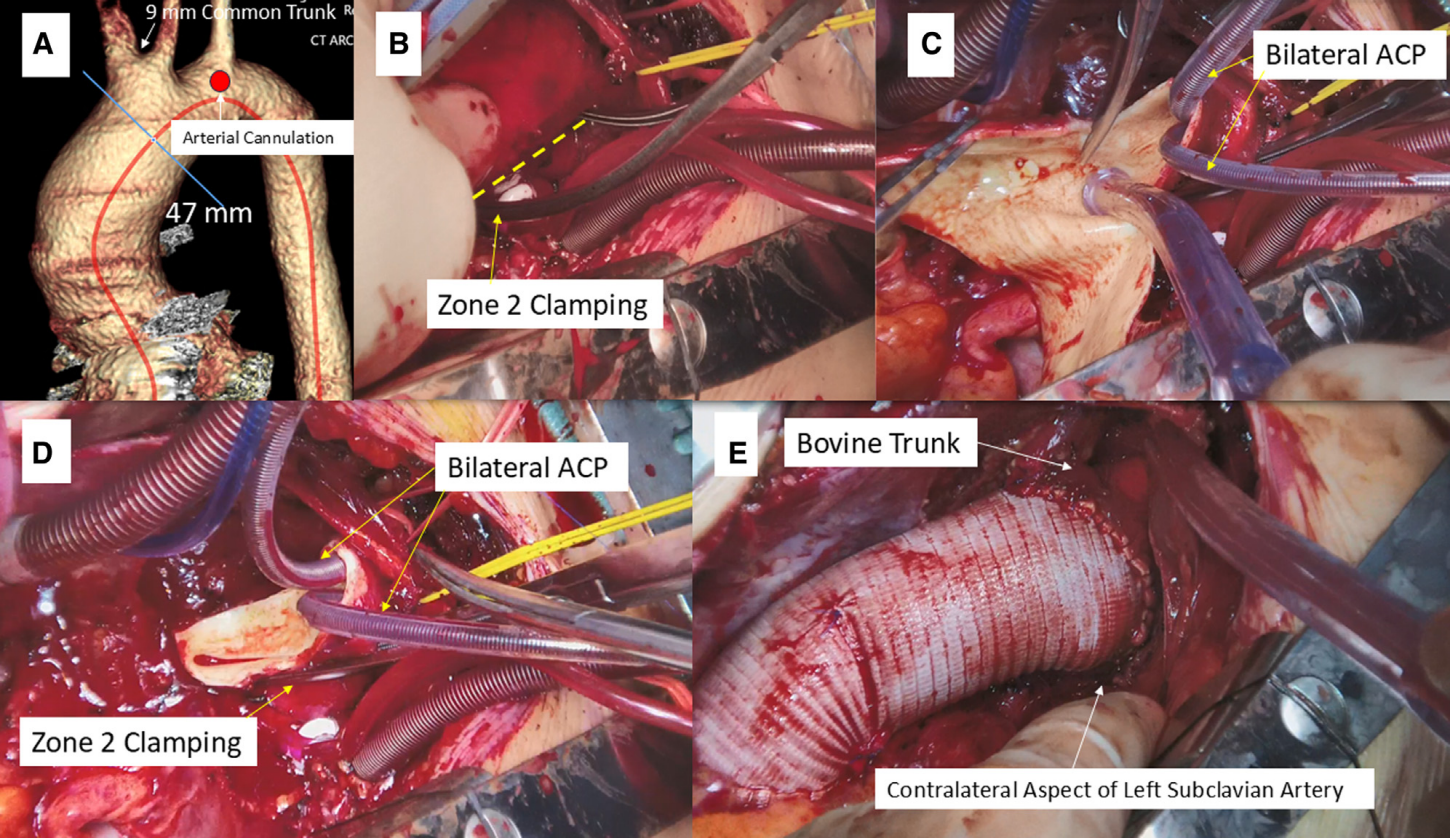
Warm hemiarch repair utilizing bilateral antegrade cerebral perfusion (*ACP*) in a patient with bovine trunk. A, Arterial cannulation site. B, Aortic arch clamping at zone 2. C, Opening the distal ascending aorta before insertion of ACP catheters. D, Establishment of bilateral ACP. E, Distal anastomosis completion.

The preferred ACP strategy at present involves the insertion of a 15Fr or 13Fr balloon-tipped catheter (Gundry Silicone Retrograde Cannula with Manual-Inflate Cuff; Medtronic Inc) through the ostium of the innominate artery after arch clamping (Figure 1, *B-E*). After arch clamping, the balloon catheter was inserted into the innominate artery followed by inflation of the balloon and cinching down the doubly wrapped vessel loop. This open ACP deployment typically takes less than a minute. ACP was terminated near the end of the anastomosis, and the catheter was withdrawn from the vessel. As the suture was tied, the vessel loop was removed to allow more vigorous backbleeding, which facilitated the flushing of any accumulated debris adjacent to the clamp site. Bilateral ACP was employed in patients undergoing zone 2 arch repair or selected patients with hemiarch/zone 1 arch repair (Figure 3, *A-E*).

#### Aortic arch clamping

It is imperative to minimize arterial flow of the cardiopulmonary bypass during arch clamp to facilitate smooth insertion in the intended arch zone, given the limited space around the arch. Conventional Crawford clamp is our preferred clamp due to its relatively thin, long, and lightly curved jaws, which are suitable for reaching the contralateral aspect of the left subclavian artery at the distal arch along the lessor curvature.

#### Zone 2 arch repair with subsequent endovascular aortic repair

**Figure E1:**
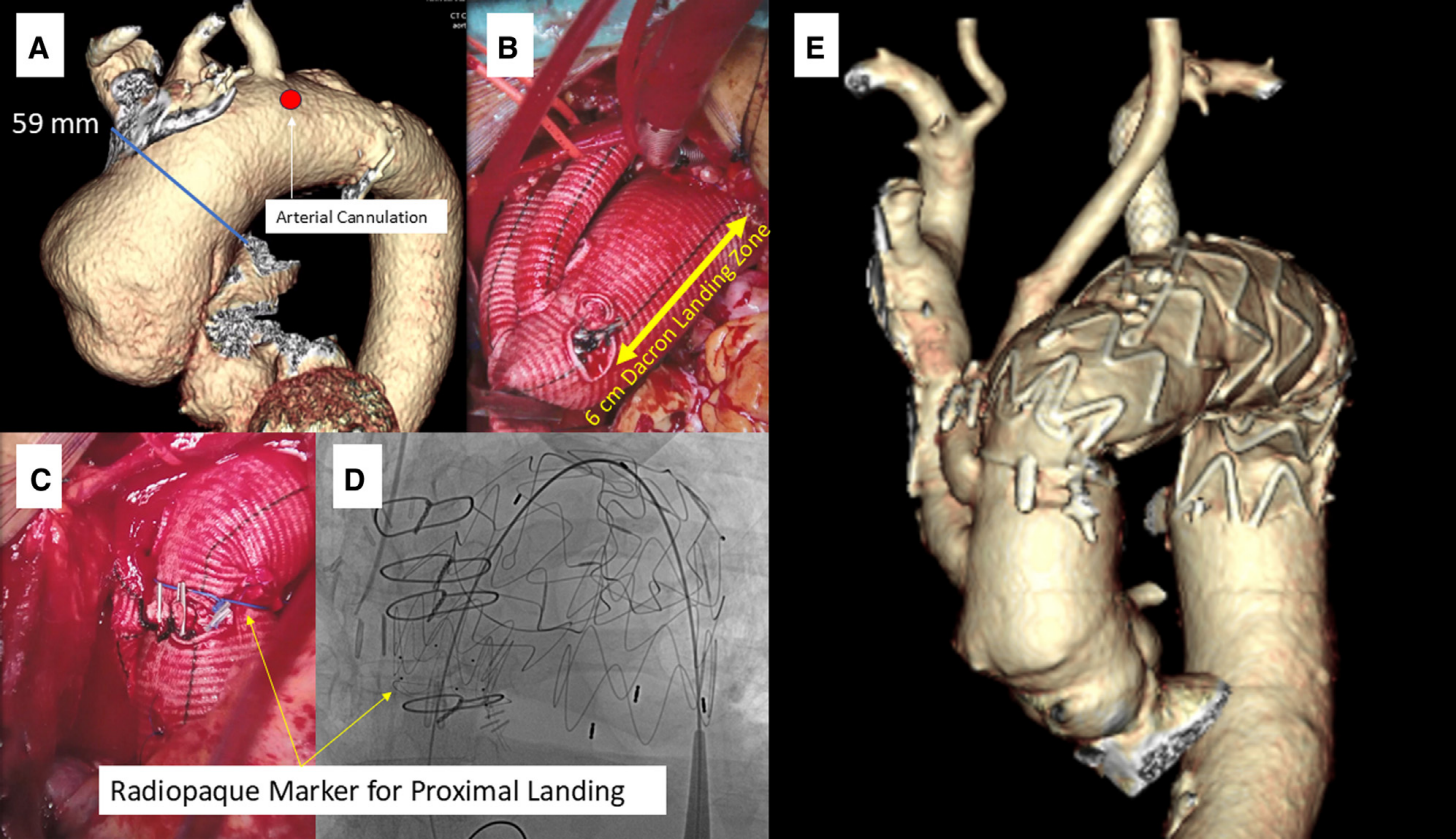
Extended aortic arch repair strategy utilizing warm zone 2 arch repair and thoracic endovascular aortic repair (*TEVAR*). A, Aortic arch aneurysm pathology needing extended aortic arch repair. B, Warm zone 2 aortic arch repair with a 6-cm polyethylene terephthalate length between the left carotid limb and the distal anastomosis to serve as a future landing zone. Polyethylene terephthalate landing zone creation. C, A pacing wire placement (*yellow arrow*) distal to the left carotid artery limb to serve as the ring radiopaque marker. D, Completion TEVAR and its proximal landing zone guided by pacing wire radiopaque marker (*yellow arrow*). E, Postoperative computed tomography scan with 3-dimensional reconstruction.

When the aortic pathology is extending beyond the arch, thoracic endovascular aortic repair is planned during the same hospitalization. In this scenario, zone 2 arch repair was modified by leaving a 3- to 6-cm polyethylene terephthalate length between the left carotid limb and the distal anastomosis to serve as a future landing zone. A pacing wire was wrapped distal to the left carotid artery limb to serve as the ring radiopaque marker (Figure E1, *A-E*).

#### Technical modifications for DeBakey type 1 aortic dissection

**Figure E2:**
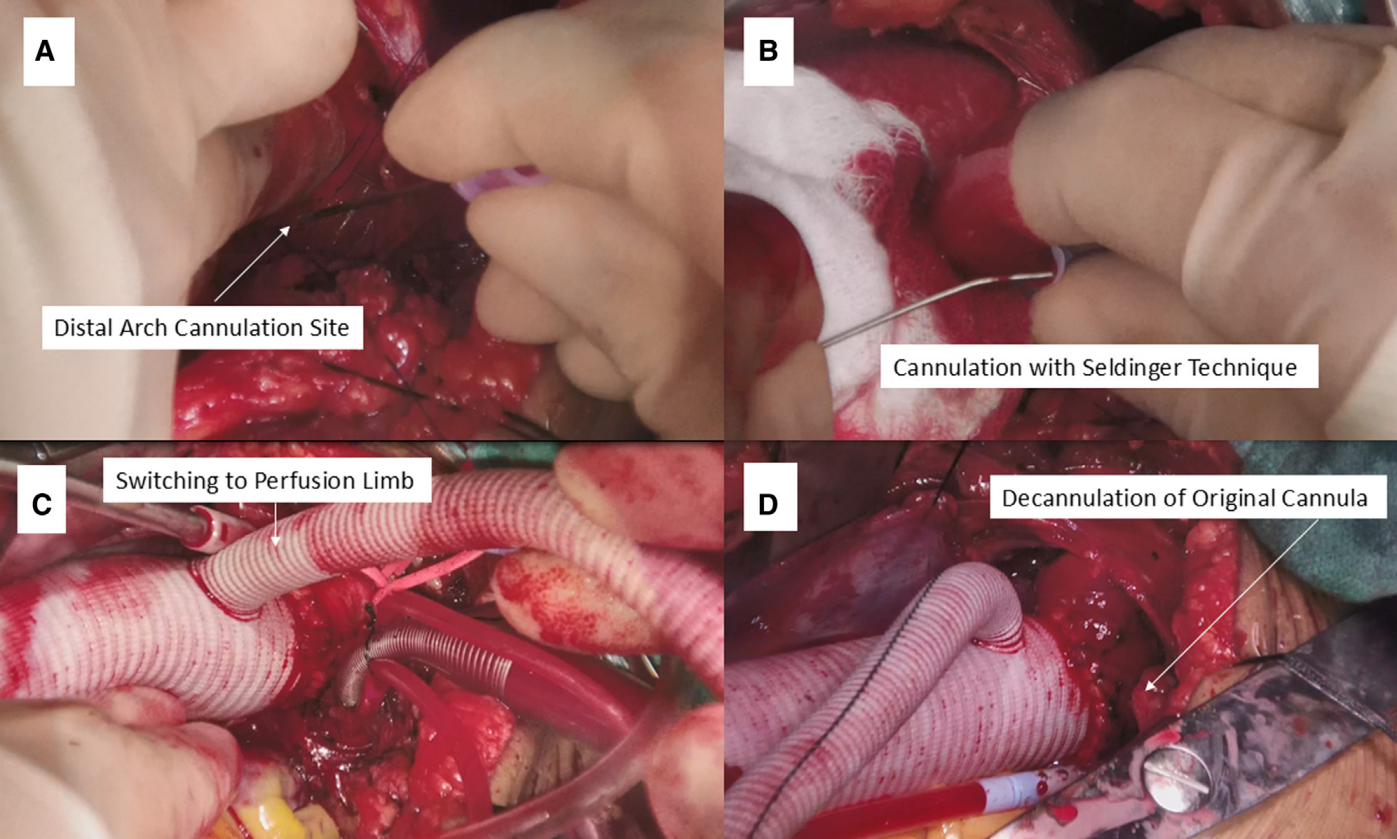
Technical modifications for DeBakey type 1 aortic dissection. A, Cannulation was performed at the anterosuperior aspect of the arch between the left common carotid and left subclavian artery after placing double purse-string sutures. B, True lumen arterial cannulation using the Seldinger technique under transesophageal echocardiography guidance. C, After distal anastomosis completion, the arterial line was switched to the side arm of the graft immediately after completing the distal anastomosis. D, The original arterial cannula was removed.

The procedure was modified following a failure due to excessive false lumen pressurization with innominate artery malperfusion after distal anastomosis completion. It was hypothesized that the distal anastomosis suture line created a blind end of the pressurized false lumen. The distal aortic arch cannula might have retrogradely perfused the false lumen through adjacent re-entry tears. To address this, the arterial line was switched to the side arm of the graft immediately after completing the distal anastomosis. Subsequently, the original central cannula was removed (Figure E2, *A-D*).

### Statistical Analysis

Continuous variables are expressed as median with interquartile range (IQR). Categorical variables are presented as proportion and absolute number. Differences between groups were detected using the χ^2^ test or Fisher exact test for categorical variables and Mann-Whitney *U* test for continuous variables. All *P* values were the result of 2-tailed tests. The statistical analyses were performed using SPSS version 27.0 (IBM-SPSS Inc).

## Results

### Trends of Warm Aortic Arch Repair

**Figure 4:**
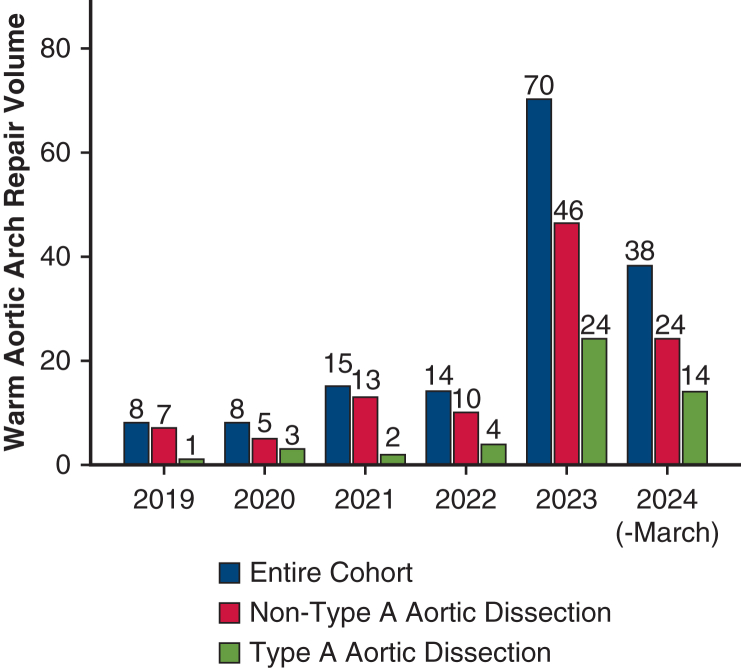
Case volume of warm arch repair during the study period.

**Figure E3:**
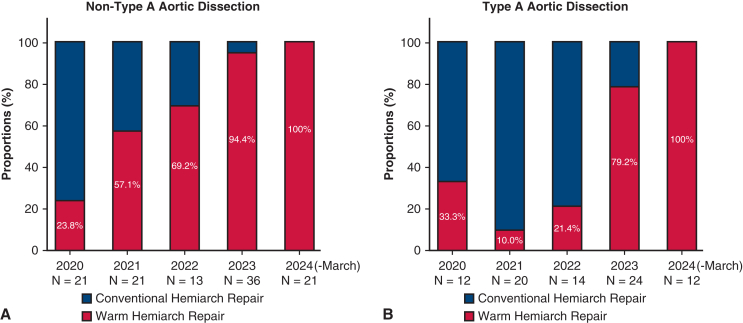
The initial experience of a single surgeon with warm hemiarch repair and its proportion among all consecutive hemiarch cases. Overall, the proportion of warm hemiarch repair exhibited an upward trend and reached 100% during 2024. A, Non-Type A aortic dissection cohort. B, Type A aortic dissection cohort.

**Figure E4:**
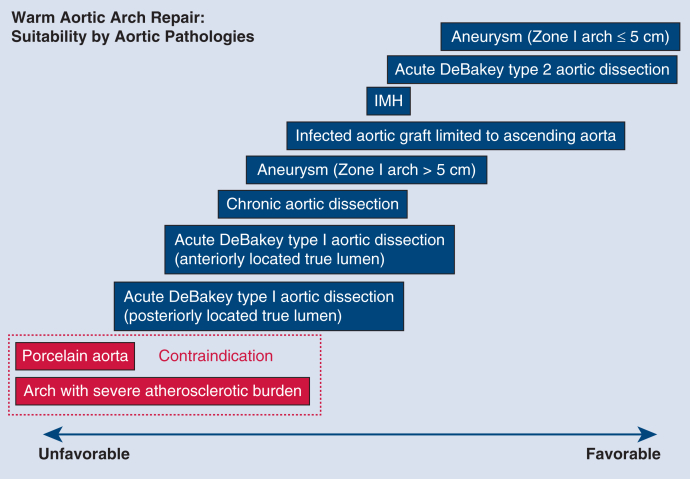
Summary of suitability for warm aortic arch repair based on aortic pathologies. *IMH*, Intramural hematoma.

The volume of warm arch repair cases increased over time from 8 in 2019 to 70 in 2023. Notably, the case volume of 38 repairs during the first quarter suggests an anticipated annual volume well above 100 in 2024 (Figure 4). Figure E3, *A* and *B*, illustrates the initial experience of a single surgeon with warm hemiarch repair and its proportion among all consecutive hemiarch cases. Overall, 143 cases out of 153 cases (93.5%) in this series were performed by the surgeon. The proportion of warm hemiarch repair exhibited an upward trend and reached 100% during 2024. The adoption of this approach began with non-aortic dissection cases, gradually encompassing Type A aortic dissection pathology more frequently in recent years. Based on our experience, the suitability of warm arch repair by aortic pathology is summarized in Figure E4.

### Patient Demographics

Table 1 presents patient demographic characteristics. Patients with non-Type A aortic dissection more frequently had a bicuspid valve, endocarditis, and prior sternotomy compared with patients with Type A aortic dissection. Bovine arch was identified in 16.3% of patients. Common previous cardiac surgeries included aortic valve replacement and aortic repair, with half of them (n = 6) being hemiarch repairs. These patients after hemiarch necessitated redo arch repair due to an infected polyethylene terephthalate graft (n = 3), dehisced suture line (n = 2), and remnant arch aneurysm (n = 1). Some patients with Type A aortic dissection were critically ill, with approximately 30% of the cohort exhibiting hemopericardium, and one-third presenting with cardiac tamponade, leading to 2 patients receiving cardiopulmonary resuscitation before repair. End-organ malperfusion was also common, observed in 29.2% of the cohort (Table E1). Additionally, 2 (4.2%) patients were receiving direct oral anticoagulants.

**Table 1:** Patient demographic characteristics

Variables	Aneurysm/other (n = 105)	Type A aortic dissection (n = 48)	*P* value
Age (y)	64 (54-72)	66 (55-76)	.24
Female sex	29 (27.6)	20 (41.7)	.084
Hypertension	77 (73.3)	39 (81.3)	.29
Diabetes	17 (16.2)	7 (14.6)	.80
End-stage renal disease on dialysis	2 (1.9)	2 (4.2)	.59
Chronic obstructive pulmonary disease	11 (10.5)	3 (6.3)	.40
History of stroke	11 (10.5)	3 (6.5)	.40
Active smoker	14 (13.3)	7 (14.6)	.84
Bicuspid aortic valve	45 (42.9)	3 (6.3)	<.001∗
Jehovah's Witness	4 (3.8)	0	.31
Syndromic heritable disease	6 (5.7)	1 (2.1)	.34
Marfan syndrome	4 (66.7)	0	.43
Loeys-Dietz syndrome	1 (16.7)	1 (100)	.29
Turner syndrome	1 (16.7)	0	1.00
DOACs intake	0	2 (4.2)	.10
Aortic arch anatomic variations			
Bovine arch	18 (17.1)	7 (14.6)	.69
Aberrant left vertebral artery	3 (2.9)	2 (4.2)	.65
Right-sided arch	2 (1.9)	1 (2.1)	1.00
Left ventricular ejection fraction (%)	55 (52-60)	58 (55-60)	.19
Active endocarditis	10 (9.5)	0	.031∗
Infected surgical aortic graft	8 (7.6)	0	.057
Max aortic diameter (mm)	51.0 (49.0-55.0)	53.0 (46.1-58.0)	.57
Aortic diameter at the innominate artery origin (mm)	46.0 (44.0-48.0)	45.0 (40.0-48.0)	.31
Previous cardiac surgery			
Root repair	6 (5.7)	0	.18
Ascending repair	12 (11.4)	0	.019∗
Hemiarch repair	6 (5.7)	0	.18
Surgical aortic valve replacement	14 (13.3)	0	.005∗
TEVAR	1 (1.0)	3 (6.5)	.041∗
CABG	3 (2.9)	0	.55
Transcatheter aortic valve replacement	0	2 (4.2)	.097
Aortic valve repair	2 (1.9)	0	1.00
Mitral repair	2 (1.9)	0	1.00
Tricuspid repair	2 (1.9)	0	1.00
Heart transplant	1 (1.0)	0	1.00

Values are presented as n (%) or median (interquartile range). *DOACs*, Direct oral anticoagulants; *TEVAR*, thoracic endovascular aortic repair; *CABG*, coronary artery bypass grafting.

∗ Statistically significant at *P* < .05.

### Operative Data

The operative data are summarized in Table 2. There was no difference in the extent of arch repair between groups. Unilateral open ACP was more frequently utilized in patients with Type A aortic dissection. Patients with non-Type A aortic dissection had longer cardiopulmonary bypass/aortic crossclamp times, and more frequently underwent concomitant procedures than those with Type A aortic dissection. Conversely, isolated hemiarch aortic repair was more common with Type A aortic dissection. Achieving a right radial arterial pressure of 50 to 80 mm Hg was consistently attained in all cases. The overall median ACP flow was 0.8 L/minute (IQR, 0.7-1.0 L/minute). Adjusted for each patient's weight, the ACP flow was 8.6 mL/kg/minute (IQR, 7.5-10.0 mL/kg/minute; range, 5-13 mL/kg/minute) and 11.3 mL/kg/minute (IQR, 9.5-14.0 mL/kg/minute; range, 7-17 mL/kg/minute) in the unilateral and bilateral ACP groups, respectively (*P* = .030). Intraoperative blood product use is summarized in Table E2. Blood transfusion rates were low, and the quantities of each blood product used were minimal, particularly in the non-Type A dissection group.

**Table 2:** Operative data

Variables	Aneurysm/other (n = 105)	Type A aortic dissection (n = 48)	*P* value
Technical success	105 (100)	47 (97.9)	.31
Extent of aortic arch repair			
Hemiarch	97 (92.4)	40 (83.3)	.090
Zone 1	2 (1.9)	3 (6.3)	.15
Zone 2	6 (5.7)	5 (10.4)	.30
ACP delivery method			
Unilateral open ACP	40 (38.1)	31 (64.6)	<.001∗
Bilateral open ACP	10 (9.5)	5 (10.4)	.86
Unilateral closed ACP	54 (51.4)	10 (20.8)	<.001∗
Bilateral closed ACP	1 (1.0)	2 (4.2)	.23
ACP time (min)	12 (9-16)	13 (10-20)	.10
ACP flow (L/min)	0.8 (0.7-1.0)	0.8 (0.6-1.0)	.33
Cardiopulmonary bypass time (min)	134 (99-176)	91 (73-123)	<.001∗
Aortic crossclamp time (min)	108 (73-149)	74 (58-98)	<.001∗
Surgical aortic graft size (mm)	28 (26-30)	28 (28-28)	.51
Redo sternotomy	30 (28.6)	0	<.001∗
Concomitant procedure	88 (84.6)	18 (39.1)	<.001∗
Aortic valve replacement	36 (34.3)	6 (12.5)	.005∗
Aortic valve repair	13 (12.4)	3 (6.3)	.25
Root repair	41 (39.0)	10 (20.8)	.027∗
Modified Bentall	19 (46.3)	2 (20.0)	.17
Valve-sparing root reimplantation	17 (41.5)	2 (20.0)	.29
Partial root replacement	2 (4.9)	6 (60.0)	<.001∗
Ross	3 (7.3)	0	1.00
CABG	20 (19.0)	0	<.001∗
Mitral repair/replacement	2 (1.9)	1 (2.1)	1.00
Tricuspid repair	2 (1.9)	1 (2.1)	1.00
Isolated hemiarch repair	11 (10.5)	19 (39.6)	<.001∗

Values are presented as n (%) or median (interquartile range). *ACP*, Antegrade cerebral perfusion; *CABG*, coronary artery bypass grafting.

∗ Statistically significant at *P* < .05.

Clinical data regarding the subgroup with isolated hemiarch repair (n = 30), of which 63.3% were Type A aortic dissections, is summarized in Table E3. There were no mortalities or strokes. The total operative time was 119 minutes (IQR, 98-131 minutes).

### Technical Success of Warm Arch Repair

Technical success was achieved in 99.4% of cases. Central cannulation for DeBakey type 1 aortic dissection was successful in all cases, regardless of the true lumen location (directly accessible true lumen: n = 19, and posteriorly located true lumen: n = 6). No aortic arch clamp site injuries were noted, including patients with Type A aortic dissection and syndromic heritable disease. As for patients with intramural hematoma, the hematoma thickness was notably less, providing a more suitable clamp site in the mid arch.

The single technical failure occurred in a patient with DeBakey type 1 aortic dissection early in the series, resulting in malperfusion of the innominate artery due to pressurized false lumen. Prompt intervention was undertaken, including division of the innominate artery at its origin, reinitiation of unilateral open ACP, and interposition grafting. Since this case, the remainder of the DeBakey type 1 aortic dissection cases have been successfully performed after the technical modification.

### Postoperative Outcomes and Follow-up

Postoperative outcomes are summarized in Table 3. The overall in-hospital mortality was 2.0%. Causes of death included neurological (n = 1) in the non-Type A dissection and multiorgan failure (n = 2) in the Type A dissection cohort. The patient with neurological death had a diffuse atherosclerotic plaque throughout the aorta and sustained multifocal ischemic injury in the left middle cerebral artery territory. In contrast, both mortalities with multiorgan failure were patients with Type A dissection requiring preoperative cardiopulmonary resuscitation. Regarding other neurologic complications, the single patient who experienced a disabling stroke in the Type A aortic dissection cohort had a recent stroke history with underlying high-grade right internal carotid artery stenosis. The single nondisabling stroke was likely the result of dissected left vertebral artery, with full neurological recovery within weeks. No reoperations for bleeding were required in the Type A aortic dissection group.

**Table 3:** Postoperative outcomes

Variables	Aneurysm/other (n = 105)	Type A aortic dissection (n = 48)	*P* value
In-hospital mortality (%)	1 (1.0)	2 (4.3)	.22
Cause of death			
Multiorgan failure	0	2 (100)	N/A
Neurological	1 (100)	0	N/A
Disabling stroke‡	1 (1.0)	1 (2.2)	.52
Nondisabling stroke‡	0	1 (2.2)	.31
Transient ischemic attack	1 (1.0)	1 (2.2)	.52
Prolonged ventilation	15 (14.4)	14 (30.4)	.022∗
Newly started dialysis†	1 (1.0)	2 (4.4)	.22
Reoperation for bleeding	3 (2.9)	0	.55
Length of stay (d)	5 (5-7)	6 (4-12)	.46

Values are presented as n (%) or median (interquartile range).

*N/A*, Not applicable.

∗ Statistically significant at *P* < .05.

† Among patients without preoperative dialysis.

‡ Newly detected postoperative stroke.

Among patients who survived to hospital discharge, the median follow-up period was 0.8 years (IQR, 0.3-1.5 years), with 98.7% survival and 100% freedom from neurological events, without any aortic reoperations. Five (3.3%) patients, representing 45.5% of patients who underwent zone 2 arch repair, underwent planned completion thoracic endovascular aortic repair (Figure E2, *A-E*).

## Discussion

The conventional proximal aortic arch operation requires circulatory arrest under hypothermia. Despite the widespread use of adjunctive cerebral protections, it inevitably accompanies varying degrees of deleterious effects for the brain, internal organs, coagulation profile, and metabolic derangements, alongside the resulting time sensitivity of the operation. In contrast, we strongly believe that there is room for refinement in the strategy of conducting arch repair differently. The concept of the warm arch repair embodies simplicity under normothermia, rendering it universally applicable and reproducible.

There have been a few reports on the application of the arch clamping technique in the literature. These have primarily comprised case reports or small series.^6^, ^7^, ^8^, ^9^ These previous experiences with hemiarch without circulatory arrest invariably involved axillary/femoral cutdown, absent perfusion to either the right or left cerebral hemisphere, and varied degrees of hypothermia. The consistent observation of absent perfusion to 1 cerebral hemisphere during arch clamping in previous reports warrants further discussion.

Our warm arch repair technique has evolved to streamline the procedure's complexity without compromising its efficacy. The closed ACP concept employing a short-tipped cannula was adopted from the ACP technique by Jassar and colleagues.^10^ In their series of 100 patients with aneurysms, ACP was exclusively delivered via a 9Fr catheter in the innominate artery under moderate hypothermia. One caveat for needle catheter placement is the risk of innominate artery dissection, hematoma, and perforating the backwall. There were inherent limitations associated with the closed ACP. One limitation was the inability to administer ACP to dissected innominate arteries. The other limitation was the interaction of the innominate artery clamp with the aortic arch clamp placed at zone 1 or 2. Our initial concern before adopting the open ACP approach originated from the interruption of the right cerebral flow under normothermia and the potential for air or debris embolization to the innominate artery between the arch clamping and ACP catheter insertion. As our experience accrues, the open ACP has demonstrated safety and reproducibility regardless of aortic pathologies. The innominate arterial backbleeding from contralateral hemisphere perfusion mitigates embolic complications and facilitates effective de-airing upon distal anastomosis completion. Moreover, the transition from arch opening to balloon ACP cannula insertion transpires within 1 minute.

Regarding clinical outcomes, the low mortality and neurological complication rates despite the high case mix index are noteworthy. Additionally, the low incidence of reoperation for bleeding and newly initiated dialysis, despite including patients with Type A dissection, likely stem from advantages related to continuously perfused end-organs and absence of hypothermia, resulting in less frequent coagulopathy. Warm arch repair is particularly advantageous for Jehovah's Witness patients, patients receiving direct oral anticoagulants, patients with kidney disease, and those with preoperative malperfusion. The low blood transfusion rates were indeed intuitive. Greason and colleagues^11^ compared on-clamp ascending aortic replacement with hemiarch replacement under hypothermic circulatory arrest in patients with a bicuspid aortic valve. This study clearly demonstrated higher transfusion rates with the hemiarch group with an odds ratio of 1.6 (*P* = .006).^11^ The 2 mortalities in the Type A dissection cohort were patients who received cardiopulmonary resuscitation en route to the operating room. The sole neurological mortality in the entire series was the 149th patient in the series of 153 cases. This patient had a history of stoke in the presence of severe aortic atherosclerotic burden, which in retrospect, constituted a contraindication for arch clamping. Careful review of preoperative imaging with particular attention to the degree of calcification, atherosclerosis, and presence of mural thrombus in the aortic arch is of critical importance.

### Limitations

This study has several inherent limitations, including its retrospective nature. This series predominantly represents a single-surgeon series, with only a handful arch repairs performed by other surgeons who recently adopted this technique.

Technical limitations include a learning curve and lack of a dedicated aortic clamp. The fundamental steps entail aortic arch cannulation and mobilization of the arch and head-vessels. However, our technique requires only minimal dissection of the arch and head-vessels—akin to what is required during conventional arch repair under circulatory arrest. Based on our experience, clamp features are integral to successful arch clamping. A crossclamp with lightly curved thin jaws, adequate length, and a flexible shaft is optimal for crossclamping the arch. The conventional Crawford clamp closely approximates these features. Our group is developing a dedicated crossclamp for warm arch repair. Central cannulation for DeBakey type 1 aortic dissection under echocardiography guidance may not be universally adopted. Six cases with DeBakey type 1 dissection underwent central cannulation in the distal arch through the false to true lumen because the true lumen was posteriorly situated. This resulted in a small septal fenestration after decannulation. None of these cannulation sites exhibited bleeding or pseudoaneurysms, likely attributable to much lower pressure within the false lumen postrepair. Furthermore, all patients demonstrated stable aortic dimensions on short-term follow-up imaging surveillance. In this context, posteriorly located true lumen does not constitute a contraindication for warm arch repair.

We have presented our early experience with warm arch repair, which is safe, simple, and reproducible without extra incisions under true normothermia. It has evolved to become a valid approach for various aortic pathologies. The development of a dedicated clamp, facilitating smoother and more intuitive jaw passage and arch clamping, is underway. Further investigations comparing the clinical outcomes and cost analysis of warm arch repair with conventional arch repair under hypothermic circulatory arrest are ongoing.

### Webcast

You can watch a Webcast of this AATS meeting presentation by going to: https://www.aats.org/resources/warm-aortic-arch-repair-withou-7071.

**Figure:**
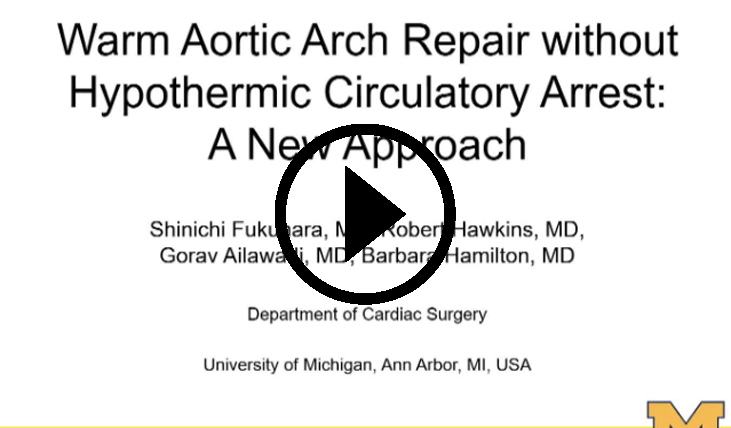

## Conflict of Interest Statement

Dr Fukuhara serves as a consultant for Terumo Aortic, Medtronic, and Artivion. Dr Ailawadi serves as a consultant/advisory board member for Medtronic, Abbott, Edwards Lifesciences, and Cephea. All other authors reported no conflicts of interest.

The *Journal* policy requires editors and reviewers to disclose conflicts of interest and to decline handling or reviewing manuscripts for which they may have a conflict of interest. The editors and reviewers of this article have no conflicts of interest.

## Figures and Tables

**Table E1:** Clinical features of patients with acute Type A aortic dissection

	Type A aortic dissection (N = 48)
DeBakey type 1 aortic dissection	32 (66.7)
Directly accessible true lumen	26 (81.3)
Posteriorly located true lumen	6 (18.8)
DeBakey type 2 aortic dissection	16 (33.3)
Intramural hematoma	8 (16.7)
Hemopericardium	17 (35.4)
Cardiac tamponade	5 (10.4)
Cardiopulmonary resuscitation	2 (4.2)
End-organ malperfusion	14 (29.2)
Cerebral	7 (50.0)
Gut	5 (35.7)
Renal	5 (35.7)
Leg	3 (21.4)
Coronary	1 (7.1)

Values are presented as n (%).

**Table E2:** Summary of intraoperative blood product use

	Aneurysm/other (n = 105)	Type A aortic dissection (n = 48)	*P* value
Any blood products used	14 (13.3)	24 (50.0)	<.001∗
PRBCs used	10 (9.5)	18 (37.5)	<.001∗
Total PRBCs (U)	31	53	< .001∗
Median PRBCs (U)	0 (0-0)	0 (0-2.0)	<.001∗
FFP used	9 (8.6)	19 (39.6)	<.001∗
Total FFPs (U)	27	52	<.001∗
Median FFPs (U)	0 (0-0)	0 (0-2.0)	<.001∗
Platelets used	13 (12.4)	21 (43.8)	<.001∗
Total platelets (unit)	34	59	<.001∗
Median platelets (unit)	0 (0-0)	0 (0-2.0)	<.001∗
Cryoprecipitate used	1 (1.0)	3 (6.3)	.092
Total cryoprecipitate (U)	1	7	.056
Median cryoprecipitate (U)	0 (0-0)	0 (0-0)	.056
Massive blood transfusions	0 (0)	0 (0)	N/A

Values are presented as n (%) or median (interquartile range). *PRBC*, Packed red blood cells; *FFP*, fresh frozen plasma; *N/A*, not applicable.

∗ Statistically significant at *P* < .05.

**Table E3:** Clinical data in patients with isolated hemiarch repair

	Isolated hemiarch repair (N = 30)
Acute Type A aortic dissection	19 (63.3)
Hemopericardium	5 (26.3)
Cardiac tamponade	2 (40.0)
End-organ malperfusion	4 (21.0)
Cerebral	3 (75.0)
Gut	1 (25.0)
Redo sternotomy	1 (3.3)
Total operative time (min)	119 (98-131)
Cardiopulmonary bypass time (min)	70 (58-84)
Aortic crossclamp time (min)	53 (44-63)
ACP time (min)	12 (10-16)
In-hospital mortality (%)	0
Neurologic complications	
Disabling stroke	0
Nondisabling stroke	0
Transient ischemic attack	1 (4.2)
Prolonged ventilation	8 (26.7)
Newly initiated dialysis	0
Reoperation for bleeding	0
Length of stay (d)	6 (5-8)

Values are presented as n (%) or median (interquartile range). *ACP*, Antegrade cerebral perfusion.

## Supplementary Data

Video 1 Standard setup and conduct of operations for warm aortic arch repair. Video available at: https://www.jtcvs.org/article/S2666-2507(25)00035-5/fulltext.
