# Supplementary figures and images for: Warm aortic arch repair: A new approach
Source: JTCVS Tech. 2025 Jan 23;31:18–31. doi: 10.1016/j.xjtc.2025.01.003 (PMC12237780; doi:10.1016/j.xjtc.2025.01.003)

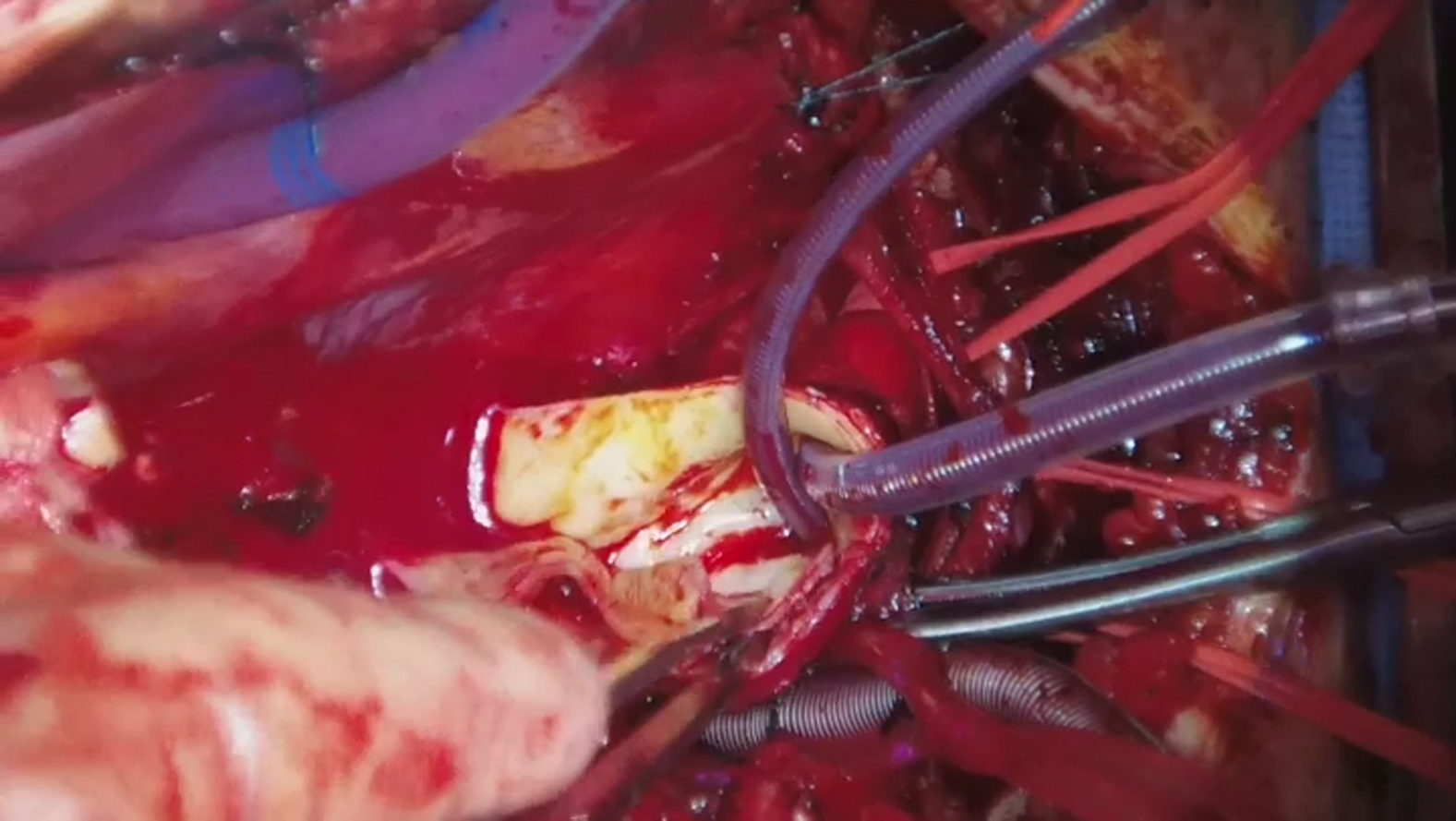

Supplement: Video 1 — Standard setup and conduct of operations for warm aortic arch repair. Video available at: https://www.jtcvs.org/article/S2666-2507(25)00035-5/fulltext. [file fx3.jpg]
